# EDITOR’S MESSAGE / MESSAGE DE LA RÉDACTION

**DOI:** 10.29173/jchla29888

**Published:** 2025-08-01

**Authors:** Jessica McEwan

**Affiliations:** JCHLA/JABSC Editor

*[Le français suit]*

Welcome to the summer issue of the Journal of the Canadian Health Libraries Association.

Here is what you’ll find in the journal’s digital pages.

## Research articles

*Demonstrating library value: the development of a customizable Library Value Planner* Mark Mueller, Nicole Askin, Jeanna Hough, Brooke Ballantyne Scott, and Joan Bartlett

*“I only knew how to Google”: students’ reflections on a four-year information literacy curriculum* Stephanie Sanger and Denise A. Smith

## Book reviews

*Cultural humility in libraries: a call to action and strategies for success* By S.D. Jones and B. Murphy (editors)

Review by: Daphne Horn

*Health literacy and libraries*

By E. Vardell and D.H. Charbonneau (editors) Review by: Kallen Rutledge

*Making the library accessible for all: a practical guide for librarians* (2nd Ed.) By Jane Vincent

Review by: Hailey Siracky

## Product Reviews

*Undermind*

Review by: Dean Giustini

An AI-powered information retrieval system described as “the Google of scientific research” which uses Semantic Scholar as its source database.

*Perplexity*

Review by: Angélique Roy

An AI-driven search engine that uses machine learning and natural language processing to source reliable information from the web and deliver sentence-form responses.

## CHLA conference abstracts

Review summaries of the papers, lightning talks, workshops, and posters from the 2025 Canadian Health Libraries Association (CHLA) conference in Vancouver.

We thank our authors and peer reviewers for their valuable contributions to the journal.

I hope you find inspiration in this latest issue.

**Jessica McEwan**

*JCHLA/JABSC Editor*

*Email:* editor@chla-absc.ca

Bienvenue au numéro estival du Journal de l'Association des bibliothèques de la santé du Canada.

Voici ce que vous trouverez dans les pages numériques de la revue.

## Articles de recherche

*Demonstrating library value: the development of a customizable Library Value Planner* Mark Mueller, Nicole Askin, Jeanna Hough, Brooke Ballantyne Scott, et Joan Bartlett

*« I only knew how to Google »: students’ reflections on a four-year information literacy curriculum* Stephanie Sanger et Denise A. Smith

## Critiques de livres

*Cultural humility in libraries: a call to action and strategies for success* Par S.D. Jones et B. Murphy (editeurs)

Critique par : Daphne Horn

*Health literacy and libraries*

Par E. Vardell et D.H. Charbonneau (editeurs) Critique par : Kallen Rutledge

*Making the library accessible for all: a practical guide for librarians* (2ème ed.) Par Jane Vincent

Critique par : Hailey Siracky

## Critiques de produits

*Undermind* Évaluation par : Dean Giustini

Un outil de recherche d'informations alimenté par l'IA, décrit comme « le Google de la recherche scientifique », qui utilise Semantic Scholar comme base de données source.

*Perplexity*

Revue par : Angélique Roy

Un moteur de recherche piloté par l'IA qui utilise l'apprentissage automatique et le traitement du langage naturel pour trouver des informations fiables sur le web et qui fournit des réponses sous forme de phrases.

## Résumés de la conférence de l'ABSC

Consultez les résumés des articles, des présentations éclair, des ateliers et des posters de la conférence de l'Association des bibliothèques de la santé du Canada (ABSC) qui a eu lieu à Vancouver en 2025.

Nous remercions nos auteurs et nos évaluateurs de pairs pour leur précieuse contribution à la revue.

J'espère que ce dernier numéro vous apportera de l'inspiration.

**Jessica McEwan**

*Directrice de la rédaction JABSC/JCHLA Courriel :* editor@chla-absc.ca

